# Novel tubular–glomerular interplay in diabetic kidney disease mediated by sirtuin 1, nicotinamide mononucleotide, and nicotinamide adenine dinucleotide Oshima Award Address 2017

**DOI:** 10.1007/s10157-019-01719-4

**Published:** 2019-03-11

**Authors:** Kazuhiro Hasegawa

**Affiliations:** Department of Internal Medicine, School of Medicine, Keio University, 35 Shinanomachi, Shinjuku-ku, Tokyo, 160-8584 Japan

**Keywords:** Sirtuin 1, Tubuloglomerular feedback, Diabetic kidney disease, Nicotinamide mononucleotide

## Abstract

Tubules interact with glomeruli, which are composed of podocytes, parietal epithelial cells, mesangial cells, and glomerular endothelial cells. Glomerular–tubular balance and tubuloglomerular feedback are the two components of the tubular–glomerular interplay, which has been demonstrated to play roles in physiological renal function and in diabetic kidney disease (DKD), in which proteins leaking from glomeruli arrive at tubular regions, leading to further tubular injury caused by the accumulation of proteinuria-inducing reactive oxygens species and various cytokines. In the current review, we present our recent work identifying a novel tubular–glomerular interplay in DKD mediated by sirtuin 1 and nicotinamide mononucleotide.

## Introduction

In this review, we summarize our studies revealing the novel roles of sirtuin 1 (SIRT1) and nicotinamide mononucleotide (NMN) in the tubular–glomerular interplay in diabetic kidney disease (DKD). First, we overview the basic functions of SIRT1 and NMN and changes i1 and NMN during DKD compared with the normal conditions. Moreover, we elucidate whether sodium–glucose cotransporter 2 (SGLT2) inhibitors retain SIRT1 and NMN function and assess the relationships among SGLT2, SIRT1, and NMN. Overall, our findings suggest the SGLT2–SIRT1–NMN axis is a potential target for diagnostic biomarkers and therapeutic approaches in DKD.

### The longevity gene sirtuin 1

**Fig. 1:**
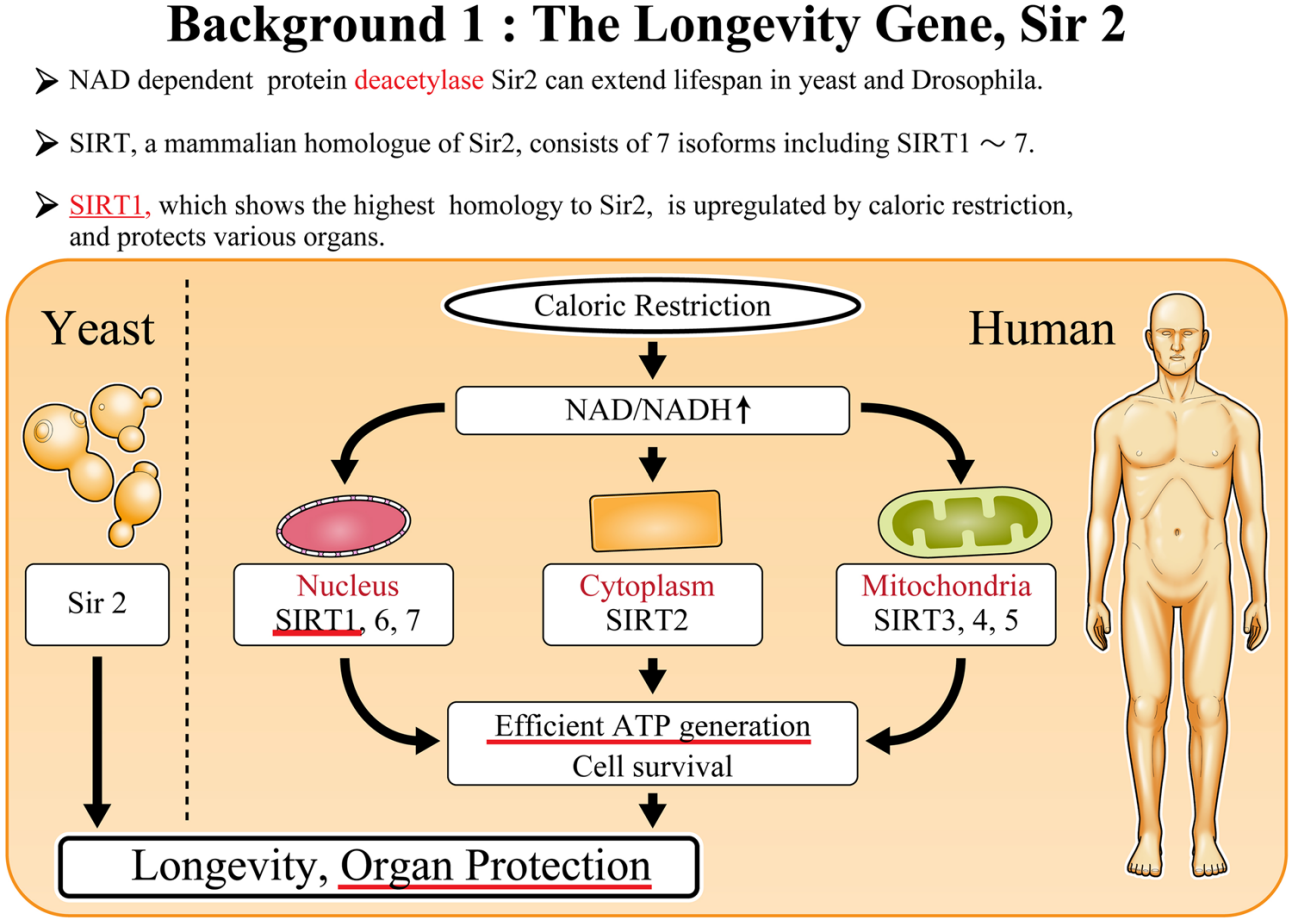
SIRT1-mediated deacetylation reaction. A variety of transcriptional factors and histones are deacetylated by SIRT1. There are two possibilities for the effects of the deacetylation of transcription factors on their activation. One is activated, and another is inactivated on a case by case basis. On the contrary, deacetylated histones cause the downregulation of their target proteins. *NAD* nicotinamide adenine dinucleotide, *Sir2* silent information regulator 2, *SIRT* sirtuin, *SIRT1* silent mating-type information regulation 2 homolog 1

We have demonstrated the role of SIRT1 in kidneys, particularly in DKD. Figure 1 outlines the basic characteristics of SIRT1, one of the seven isoforms of mammalian sirtuins, which are found in specific intracellular compartments. The first sirtuin that was discovered was Sir2 in yeast [1]. Thereafter, several studies have disclosed the important role of sirtuins in yeast as well as in higher organisms. SIRT1, SIRT6, and SIRT7 are localized in the nucleus [2]; SIRT2 is detectable in the cytoplasm [3]; and SIRT3, SIRT4, and SIRT5 are distributed in the mitochondria [4]. As a shared feature, sirtuins are upregulated by caloric restriction and elevated oxidized/reduced nicotinamide adenine dinucleotide (NAD), leading to an efficient, sirtuin-mediated ATP generation and cell survival, thereby linking sirtuins to organ protection and longevity.

## Basic functions of SIRT1

**Fig. 2:**
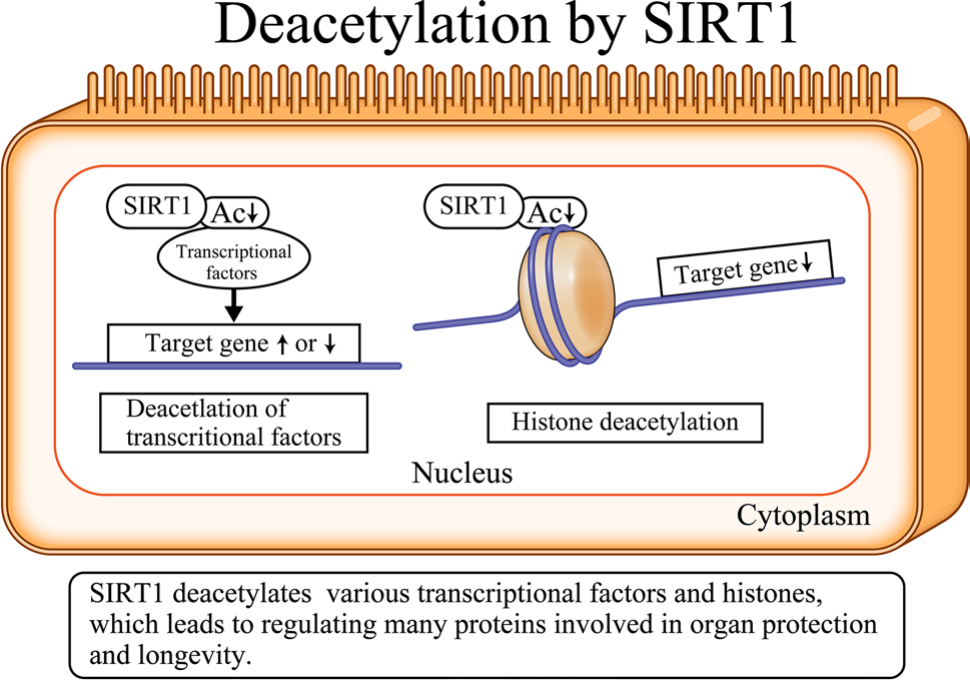
Basic function of the anti-aging gene, Sir2. Sir2 was discovered and identified in yeast. The mammalian homologue, Sirts, are composed of seven isoforms from SIRT1–7. Sirts shows different intracellular localization, but work as a protective molecules controlling longevity in a common way. *Ac* acetyl

Sirtuins are nuclear deacetylating enzymes (Fig. 2) that serve two purposes. First, several transcriptional factors are deacetylated by SIRT1 [5], and downstream target genes are upregulated or downregulated depending on the transcription factor. Moreover, SIRT1 deacetylates histone lysine residues, which leads to the transcriptional downregulation of downstream target genes.

### NAD-related metabolic maps

**Fig. 3:**
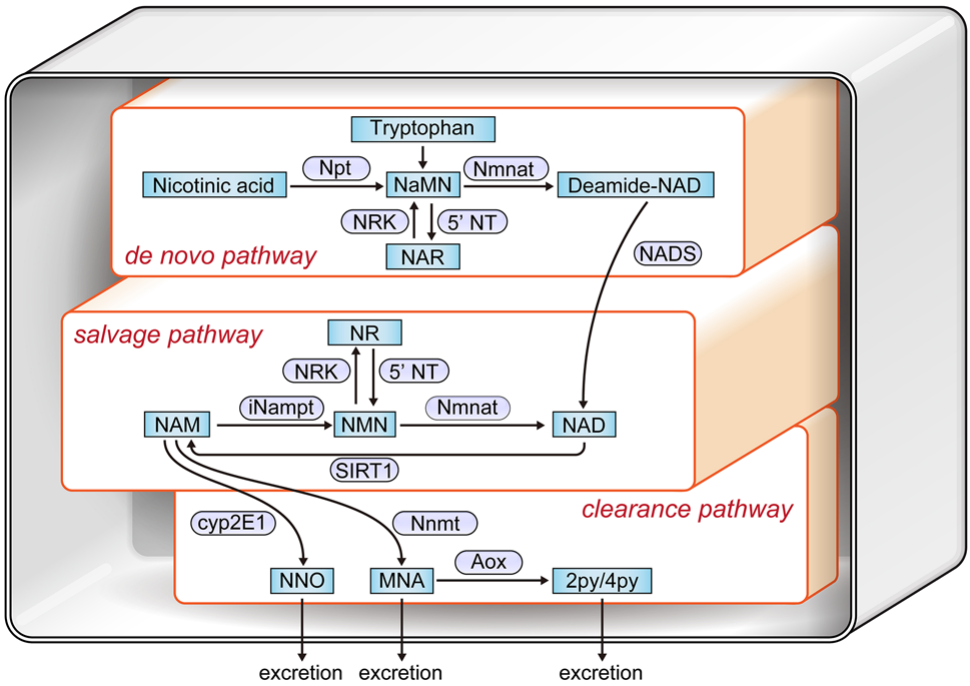
NAD-mediated metabolic map. NAD is synthesized by two main pathways, including de-novo and salvage pathways. *NAM* nicotinamide, *Npt* nicotinic acid phosphoribosyltransferase, *5′-NT* 5′-nucleotidase, *NaMN* nicotinic acid mononucleotide, *NR* nicotinamide riboside, *NMN* nicotinamide mononucleotide, *iNampt* intracellular NAM phosphoribosyl transferase (iNampt), *NRK* nicotinamide riboside kinases, *Nmnat* nicotinamide mononucleotide adenylyl transferases, *NADS* NAD synthetase, *NAR* nicotinic acid riboside, *5′-NT* 5′-nucleotidase, *Aox* aldehyde oxidase. The two end-products of Aox, N1-methyl-2-pyridone-5-carboxamide (2py) and N1-methyl-4-pyridone-3-carboxamide (4py), together with MNA are excreted in the urine. Nnmt (nicotinamide *N*-methyltransferase) converts NAM to N1-methylniacinamide (MNA). NAM is also converted by CYP2E1 (Cytochrome P450 2E1) to NNO (NAM N-oxide), which is also eliminated in the urine

Following SIRT1-mediated deacetylation of transcription factors or histones, NAD is converted into nicotinamide, as shown in the NAD-related metabolic maps in Fig. 3, thereby emphasizing that NAD is indispensable for SIRT1 function. In mitochondria, NAD is converted to NADH. Therefore, it is essential that these pathways remain intact to maintain cellular NAD levels, and hence, NAD is supplied via de novo and salvage pathways [6]. In the de novo pathway, the essential amino acid tryptophan is converted to NAD, whereas in the salvage pathway NAD consumed by SIRT1 is recycled via two enzymes—nicotinamide phosphoribosyltransferase (NAMPT) and NMN adenylyltransferase (NMNAT). NMN is a precursor of NAD that was reported to be beneficial in several diseases by restoring NAD levels. However, the reason for NMN but not NAD being more effective in elevating NAD levels remains unknown and requires further investigation. Nicotinamide, a substrate of intracellular NAMPT in the NAD-related salvage pathway, is excluded to the extracellular compartment.

### Tubular metabolic changes may precede changes in glomeruli and podocytes in DKD

**Fig. 4:**
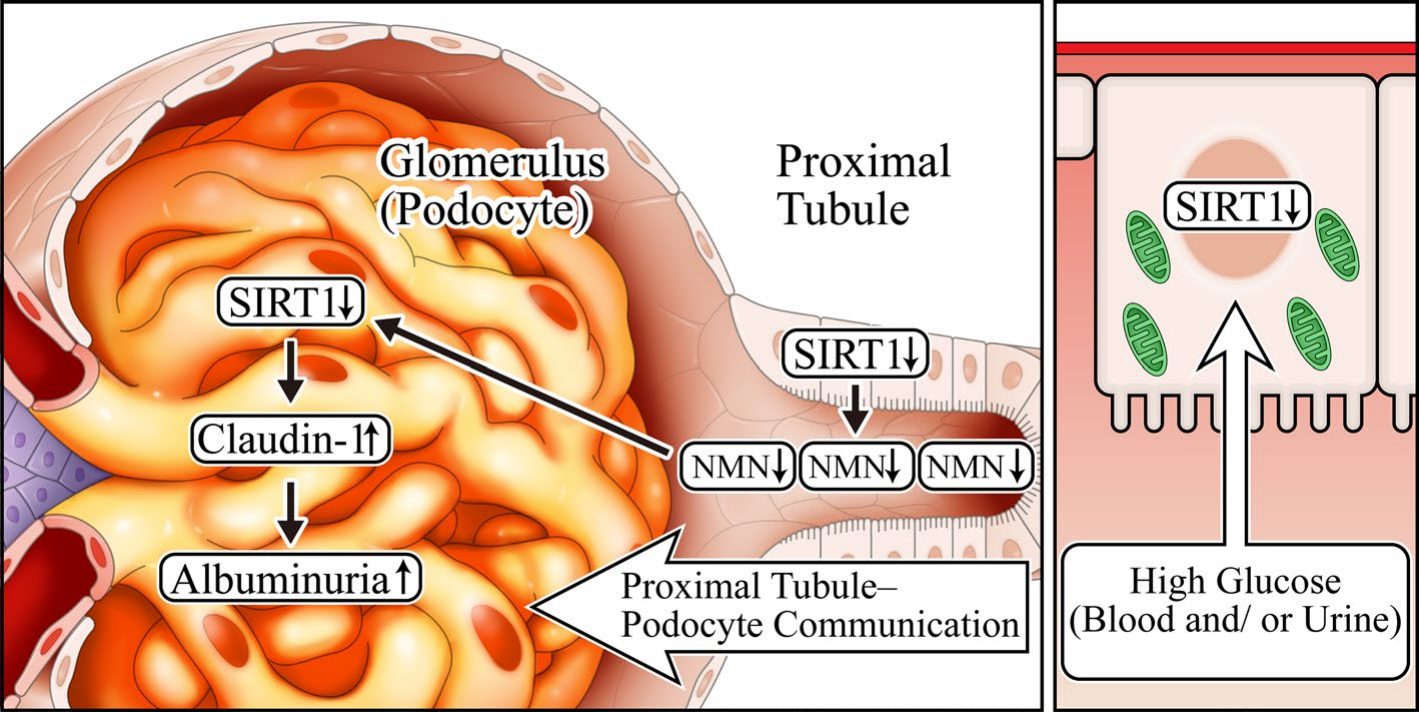
Schema depicting proximal tubule–podocyte communication. High glucose exposure primarily downregulates proximal tubular SIRT1 levels. This reduces NMN levels and podocytes’ SIRT1 levels, accompanied by claudin-1 induction and diabetic albuminuria

Previous research has revealed the pivotal role of podocyte injury in DKD [7]. Microalbuminuria is considered as an important early diagnostic marker for DKD and indicates functional or morphological damage to the podocytes. However, microalbuminuria is not a specific diagnostic marker for diabetic renal damage in DKD because it is increased in hypertension-induced renal sclerosis as well. Our recent research has revealed that metabolic changes (Fig. 4) in proximal tubules precede the changes in podocytes in DKD [8]. Specifically, proximal tubular SIRT1 was primarily downregulated before evident podocytic injury, thereby leading to a reduction in NMN levels that causes further downregulation of SIRT1 in podocytes. In the NAD salvage pathway, NMN is upstream of NAD and SIRT1. However, the salvage pathway is a circular metabolic pathway and not a linear one. It remains possible that NMN is upstream as well as downstream of SIRT1. In a previous study, we have determined whether NMN was the humoral factor connecting proximal tubules with podocytes using three approaches: experiments utilizing conditioned medium, measurement of endogenous NMN levels, and exogenous treatment with fluorophore-tagged NMN to investigate NMN biodistribution [8, 9]. We termed this cell–cell interplay as the proximal tubule–podocyte communication. As shown in later figures, the two well-known components of the tubuloglomerular communication are tubuloglomerular feedback (TGF) and glomerular–tubular balance (GTB) [10]. Corruption of the TGF and GTB underlies glomerular hyperfiltration observed in DKD. However, our new evidence suggests that the dysfunction in the proximal tubule–podocyte communication occurs prior to a malfunction in TGF and GTB and the podocyte injury.

In podocytes, SIRT1 levels are decreased, which was affected by the proximal tubular SIRT1 downregulation, thereby leading to the ectopic expression of claudin-1 in podocytes, causing albuminuria. Under normal conditions, claudin-1 is expressed in parietal epithelial cells, which creates tight junctions that might prevent leakage from Bowman’s capsule [11]. Furthermore, a recent report in mice overexpressing claudin-1 specifically in podocytes has demonstrated that podocyte damage occurred via reduced nephrin and podocin [12]. Although the authors have not elucidated the exact mechanism for the reduction of nephrin and podocin by claudin-1, they inferred that claudin-1 overexpression competitively inhibited the expression of both nephrin and podocin. These mechanisms should be investigated in future studies.

### SIRT1-mediated epigenetic regulation of claudin-1 expression in kidneys

**Fig. 5:**
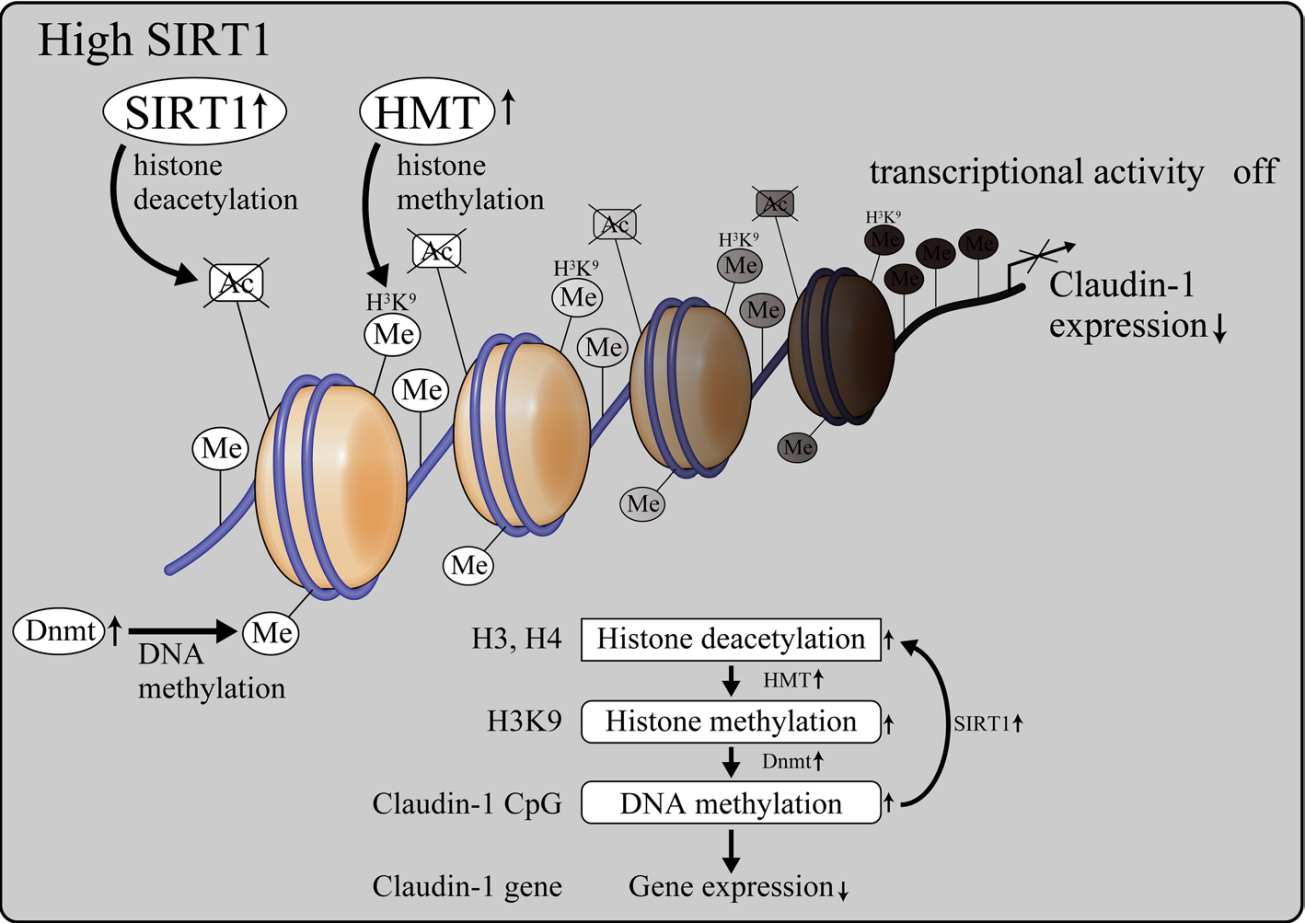
The role of SIRT1 in podocytes under normal conditions. Under normal glucose conditions, retained SIRT1 deacetylates H3 and H4 histones, resulting in H3K9 histone methylation and Dnmt1 activation. *H3K9* histone H3 Lys^9^, *HMT* histone methyltransferases, *ME* methyl-, *DNMT* DNA methyltransferases, *CG* CpG islands

**Fig. 6:**
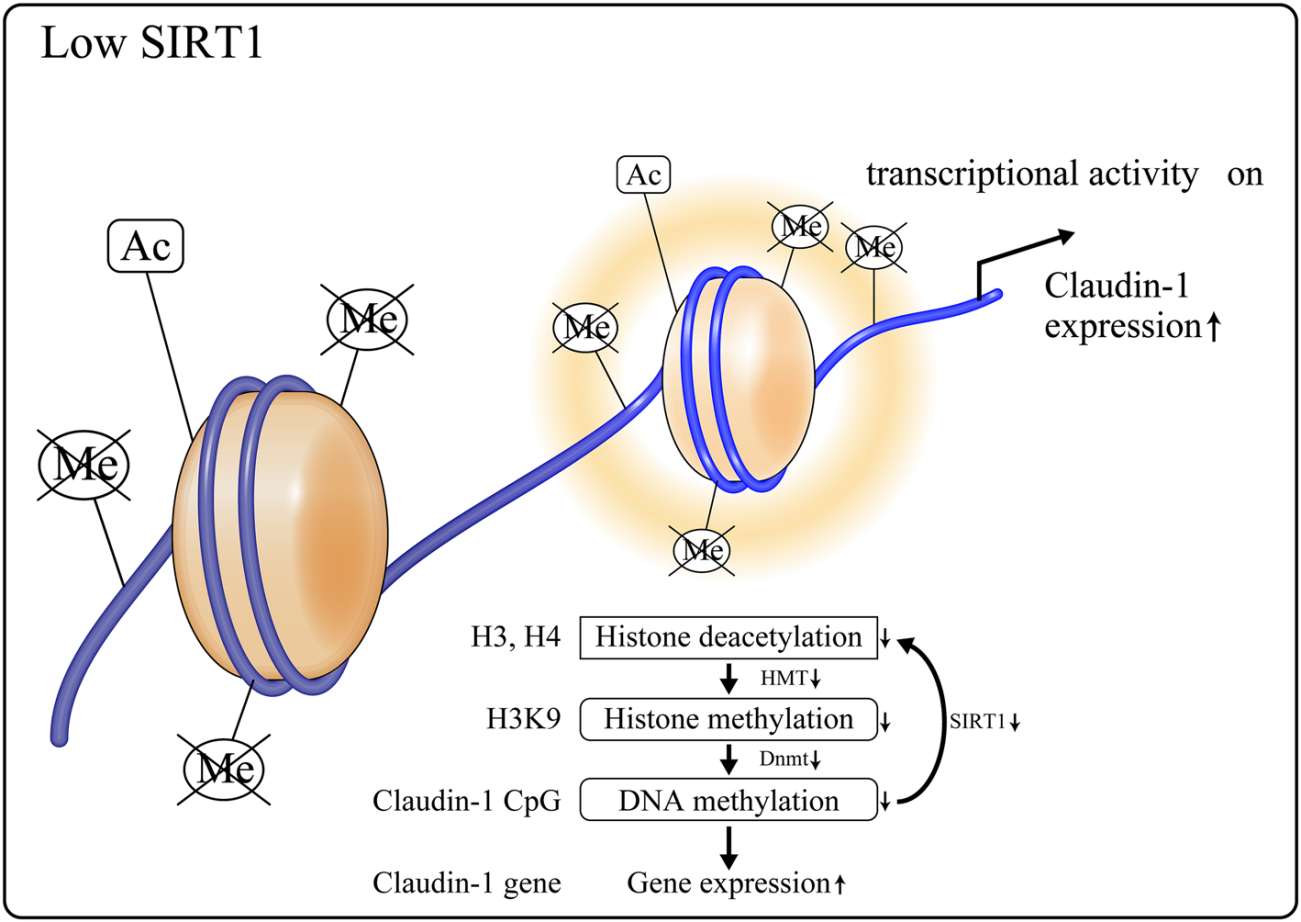
The role of decreased SIRT1 under diabetic condition in podocytes. Lowered SIRT1 elevates acetylation on H3 and H4, leading to a decrease in the histone methylation of H3K9. Thus, Dnmt1 is activated, which also reduces the DNA methylation of claudin-1 CpG islands, inducing claudin-1 expression

Our studies assessing the mechanism of SIRT1-mediated control of claudin-1 expression have suggested epigenetic mechanisms are involved in this regulation. Under normal conditions, SIRT1 levels were maintained (Fig. 5). SIRT1 deacetylated histones H3 and H4; this caused histone H3K9 methylation and DNA methylation of the CpG islands around claudin-1 gene by DNA methyltransferase (DNMT) 1, thereby suppressing claudin-1 expression. Conversely, in diabetic conditions, SIRT1 levels are reduced with the consequent elevation of H3 and H4 acetylation (Fig. 6), which in turn suppressed H3K9 methylation and DNMT1-induced CpG methylation of claudin-1 to promote its expression.

### DNMT1 is involved in the regulatory mechanism of claudin-1

**Fig. 7:**
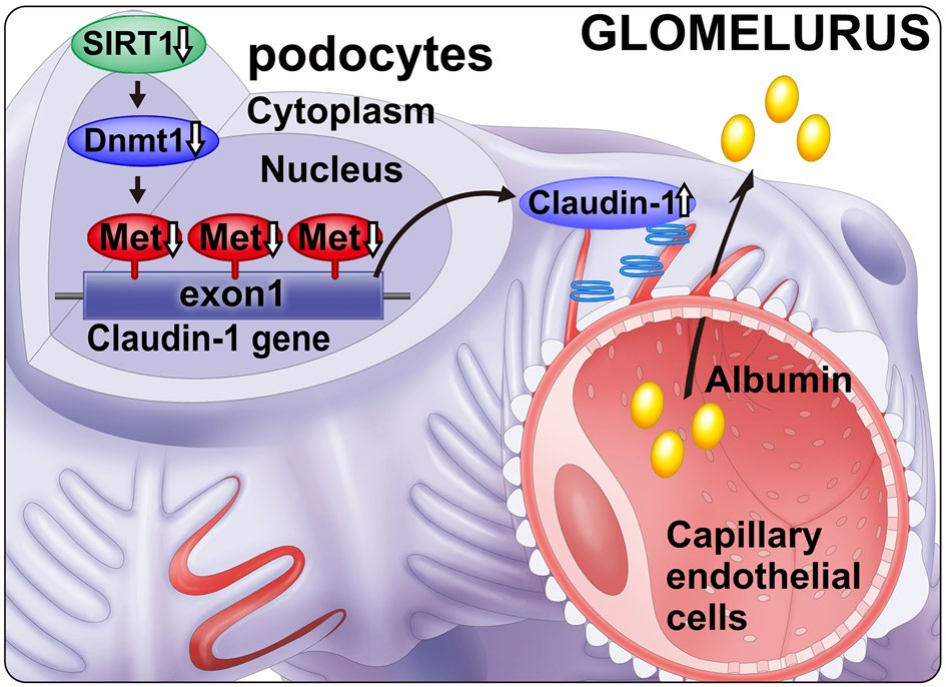
Epigenetic regulation of claudin-1. Reduced SIRT1 inactivates Dnmt1, leading to the hypomethylation of claudin-1 CpG islands. Thus, claudin-1 expression is elevated, causing diabetic albuminuria

We have determined that among the three DNMT isoforms, DNMT1 plays an important role in CpG methylation of claudin-1 (Fig. 7). We have specifically demonstrated that DNMT1 is involved in the methylation of the CpG islands located in the first exon and not of those in the promoter region of claudin-1. The CpG islands are typically localized in the promoter regions, and our findings regarding DNMT1-mediated claudin-1 methylation is unique. However, a recent report [13] has stated that first exon CpG islands played an important role in gene transcription in addition to those in promoter regions [14]. Therefore, future studies will be ciritical to further elucidate the role of exon CpGs.

### Detailed molecular mechanisms underlying the proximal tubule–podocyte interplay

**Fig. 8:**
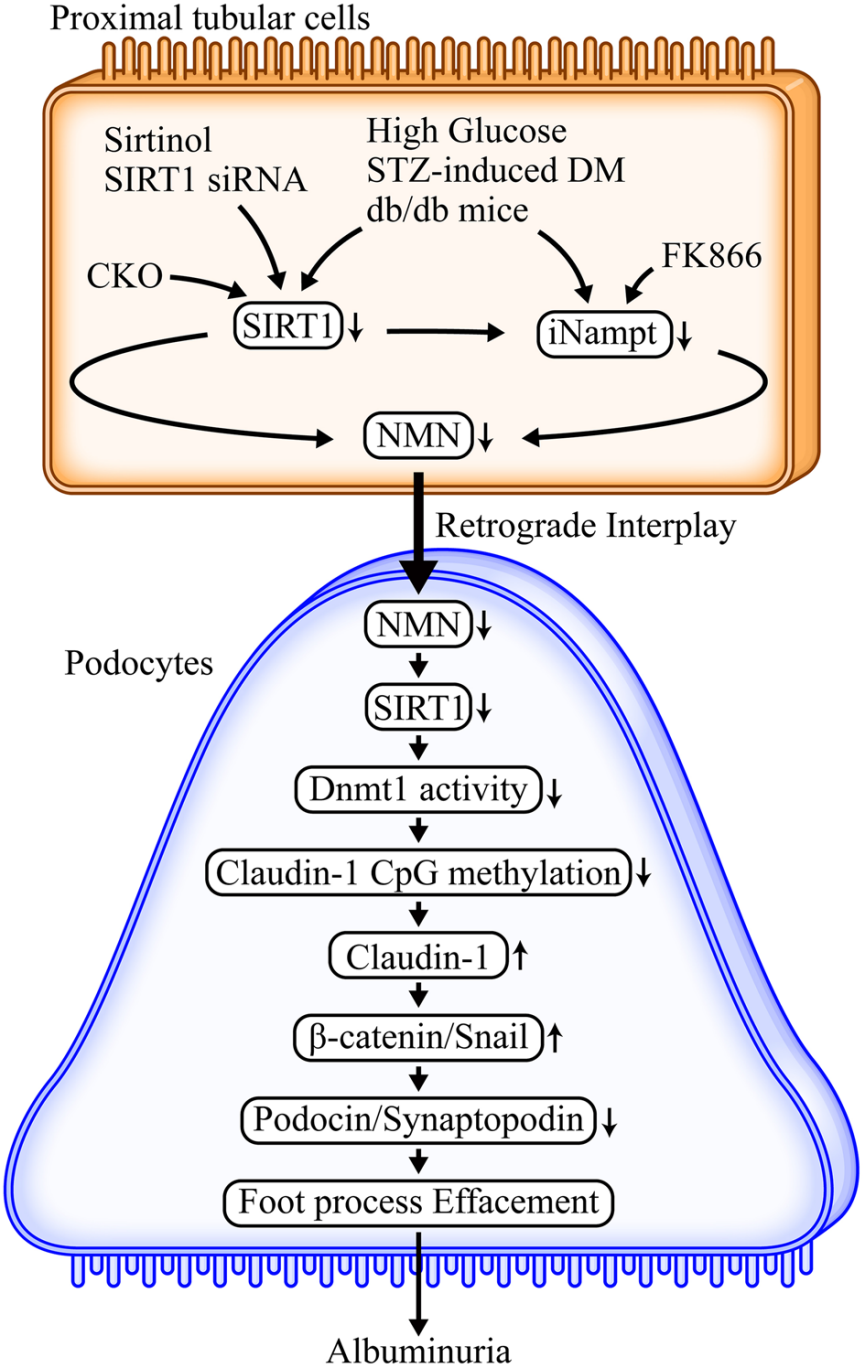
Detailed molecular mechanisms of collapsed proximal tubule–podocyte communication under diabetic nephropathy. Initial metabolic changes occurred in proximal tubules, where proximal tubular SIRT1 is reduced by high glucose exposure. This leads to the reduction in Nampt and NMN, causing the decline in the decreased SIRT1 in podocytes. Conditional knockout (CKO) mice: SIRT1 CKO, FK866: Nampt-specific inhibitor. *STZ* streptozotocin, C57BLKS/J Iar-+Lepr^db^/+Lepr^db^(db/db) mice

The detailed molecular mechanisms of claudin-1 damage in podocytes involving the β-catenin/snail pathway is shown in Fig. 8. We used DKD models, such as the streptozotocin-induced diabetes mellitus model and db/db mice, to demonstrate the corruption of proximal tubule–podocyte interaction including a reduction in proximal tubular SIRT1 levels concomitant with decreased SIRT1 and increased claudin-1 in podocytes. Additional experiments using proximal tubule-specific *Sirt1* conditional knockout mice showed phenotypes that were similar to that observed in diabetic animal models [8]. These data indicated that proximal tubular SIRT1 was a critical molecule in proximal tubule–podocyte communication. In contrast, proximal tubule-specific *Sirt1* transgenic mice rescued streptozotocin- and db/db-induced albuminuria, thereby validating the pivotal roles of SIRT1 in proximal tubules. Although we have previously demonstrated that proximal SIRT1 protected against reactive oxygen species-mediated kidney injury [15–17], we recently uncovered that SIRT1 was specifically protective against hyperglycemia and diabetes-induced kidney damage [8]. Another report by Inagi and colleagues [18] has demonstrated that podocyte-specific *Sirt1* knockout provoked podocyte damage in mice, further supporting the ciritical role of SIRT1 in protection against kidney disease. A recent review on the pivotal roles of surtuins in kidney disease [19] raise the possibility of sirtuins as novel diagnostic and/or therapeutic targets of DKD.

### SGLT2 is involved in proximal tubules and podocyte communication in DKD

**Fig. 9:**
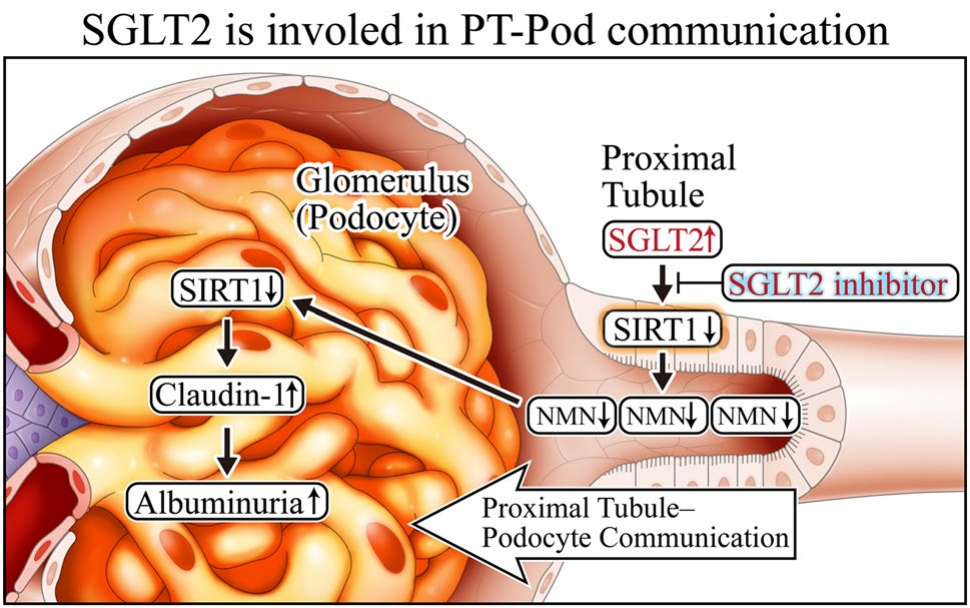
SGLT2 is involved in PT (proximal tubules)-Pod (podocyte) communication. Augmented SGLT2 expression diminishes SIRT1 expression, which is blocked by a SGLT2 inhibitor. *SGLT2* sodium-glucose cotransporter-2

Finally, we summarize the relationship between SGLT2 and SIRT1 (Fig. 9). In our recent study, we have uncovered that SGLT2 is elevated during early stages of DKD, which could upregulate intracellular glucose levels in proximal tubules and subsequently decrease SIRT1 expression. Conversely, we demonstrated that SGLT2 inhibitors preserved SIRT1 expression. Our current studies [20] focus on elucidating whether SGLT2 inhibitors can maintain the proximal tubule–podocyte communication.

### Glucose reabsorption in proximal tubules

**Fig. 10:**
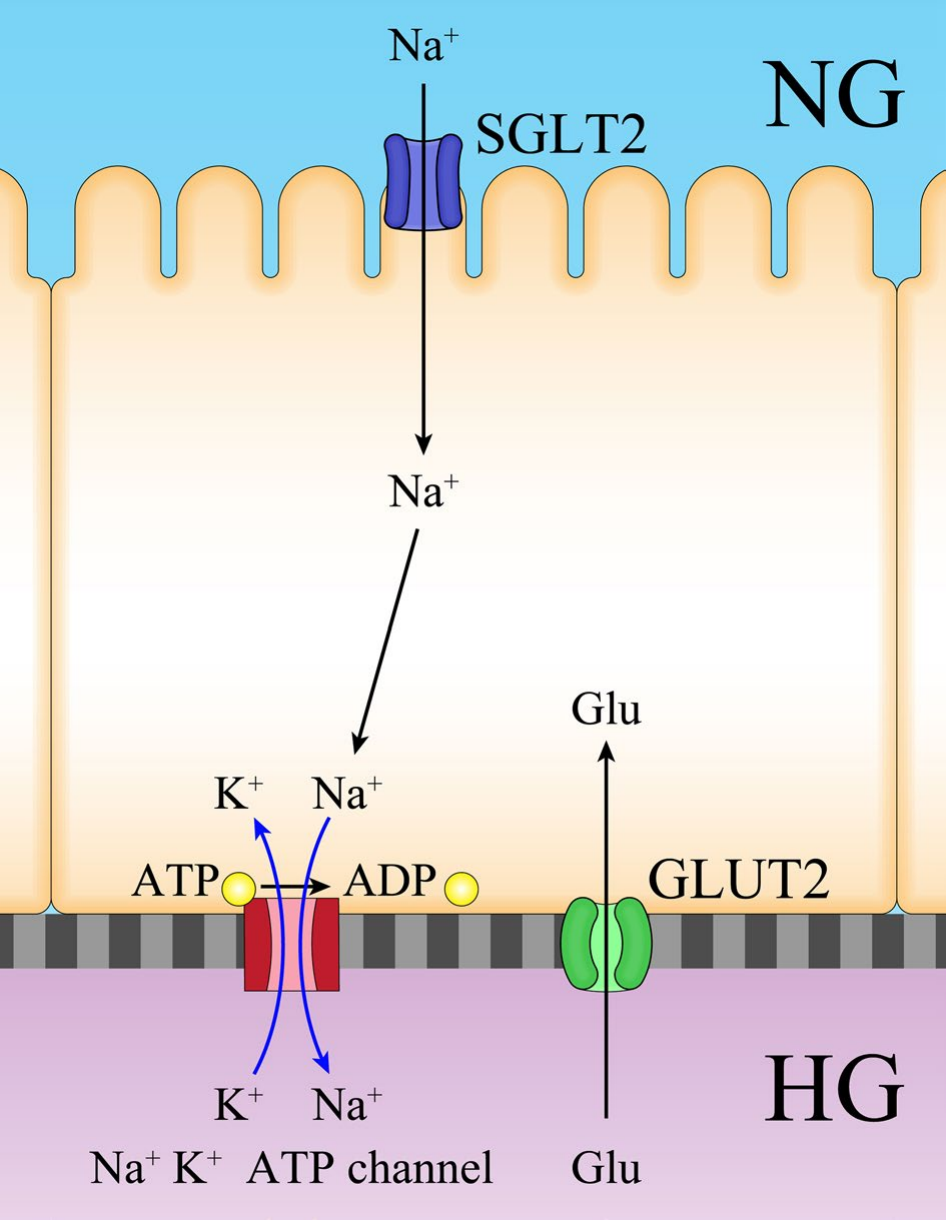
Possible hypothesis of glucose regurgitation in hyperglycemia and normal glucose urea levels. Renal glucose reabsorption occurs in proximal tubule by the coordinated action of the SGLT2 and GLUT2 located in the luminal and basolateral membranes, respectively. We hypothesize that GLUT2 is involved in the sensoring of high glucose flow in a certain condition as shown in this figure. *GLUT2* glucose transporter 2

Glucose reabsorption in proximal tubular cells is depicted in Fig. 10. Briefly, Na^+^ and glucose transports into proximal tubules via SGLT2, whereas Na^+^/K^+^ ATPase-coupled channels pump out Na^+^. Glucose is transported via glucose transporter 2 (GLUT2). Contrary to the physiological regulation of glucose, we hypothesized that GLUT2-mediated regurgitation occurs, particularly in very early stages of DKD. Further, this could occur in the stages of the disease with increased urinary glucose when hyperglycemia might not be observed because of compensatory hyperinsulinemia.

### GLUT2-mediated upregulation of SGLT2

**Fig. 11:**
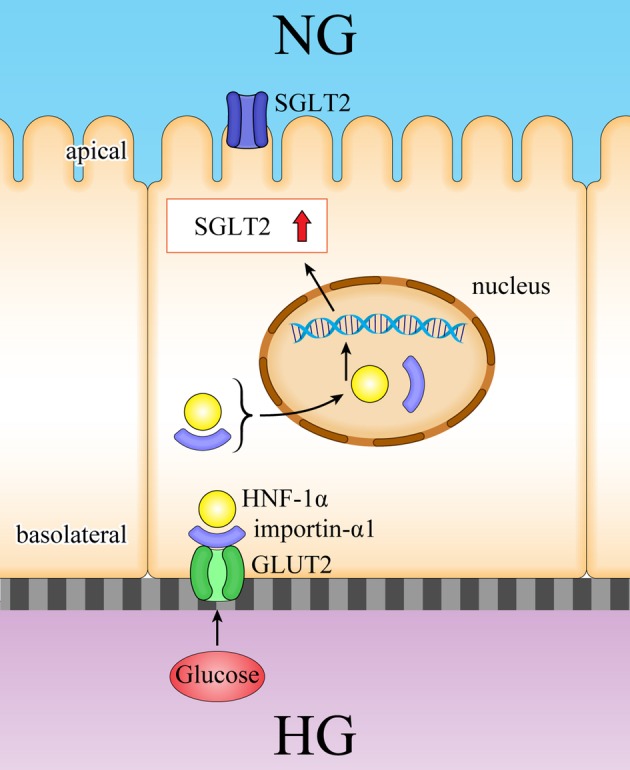
Our findings of the induction of SGLT2 expression via GLUT2/importin-α1/HNF-1α. SGLT2 was increased in the diabetic kidneys, where basolateral glucose exposure activates GLUT2/importin-α1/HNF-1α pathways. *HNF-1α* hepatocyte nuclear factor-1 homeobox A

Using a two-chamber model [20], we found that high glucose levels in the basolateral side of proximal tubules (Fig. 11) might trigger intracellular signal transduction, leading to SGLT2 upregulation. We determined that GLUT2 was coupled with importin-α1, which was bound to HNF1α. We further elucidated that after detecting glucose flow, the importin-α1/HNF1α complex was then unbound from GLUT2, and HNF1α transported into the nucleus by importin-α1 to increase the activity of the *SGLT2* promoter.

### Role of GLUT2-mediated SGLT2 upregulation in DKDs

**Fig. 12:**
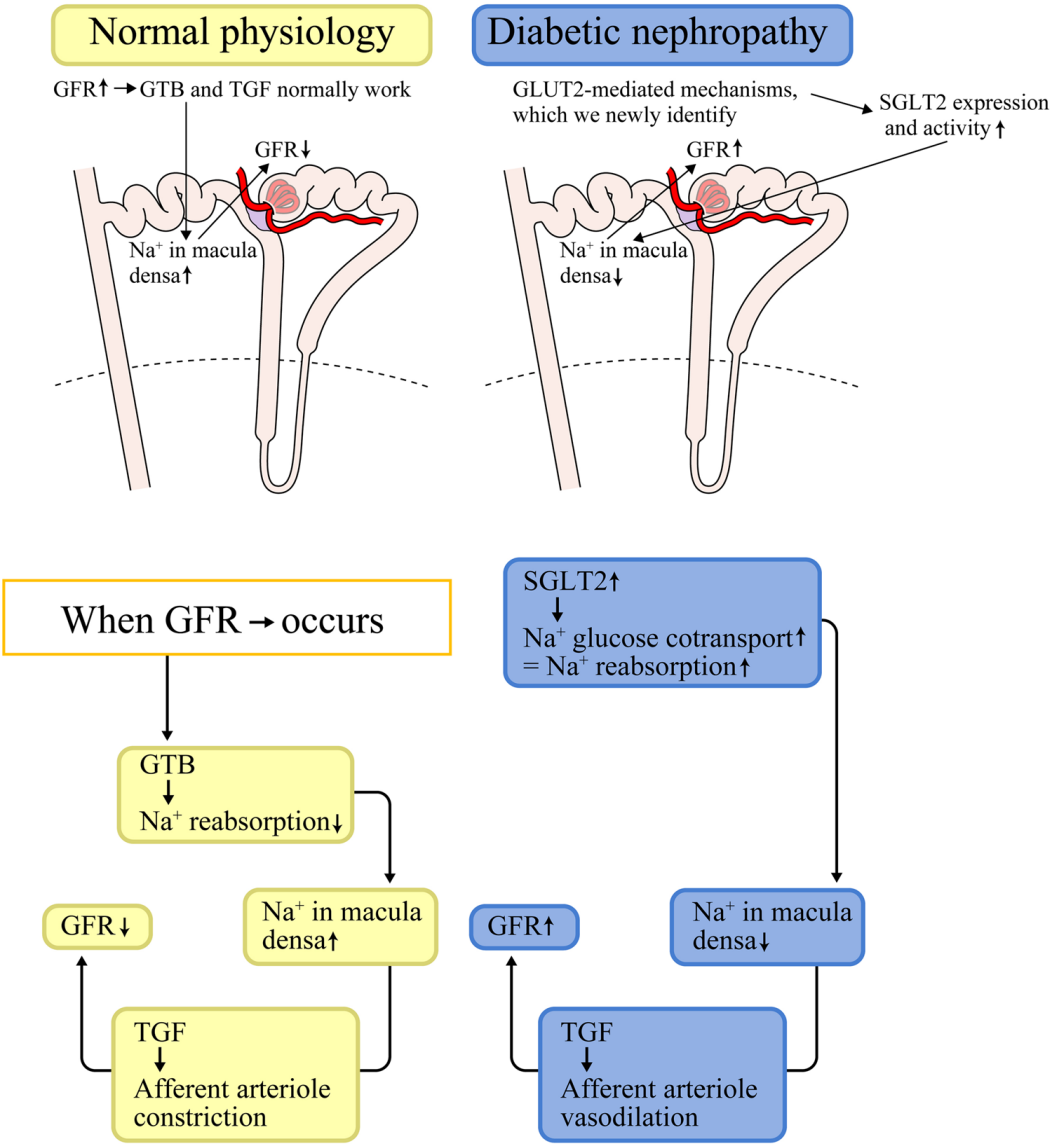
Regulatory mechanisms for GFR in comparing between normal conditions and diabetic conditions. Tubuloglomerular feedback (TGF) regulates tubular flow by detecting and correcting changes in GFR (glomerular filtration rate). Glomerulotubular balance (GTB) regulates proximal tubular reabsorption influenced by GFR

In DKD, disruption of the GTB and TGF leads to the hyperfiltration and increase in Na^+^ reabsorption. The mechanisms we uncovered imply that SGLT2 inhibitors might be utilized as a novel therapeutic target in DKD (Fig. 12) to block the disruption of GTB and TGF. Blocking the GLUT2/importin-α1/HNF1α interaction might reduce SGLT2 expression and prevent the reduction in SIRT1 expression, which may further contribute to the protection of the proximal tubule–podocyte communication in DKD.

## Conclusions and perspectives

In DKD, SGLT2 upregulation was caused by GLUT2-mediated intracellular signaling, and intracellular, SGLT2-induced high glucose levels might decrease SIRT1 expression. However, the mechanism underlying the reduction in SIRT levels downstream from increased SGLT2 require further investigation. Glucose is not metabolized in proximal tubules, which do not use glucose as a fuel; fatty acid oxidation is the main metabolic pathway for ATP generation. Therefore, glucose passes through proximal tubules, and high glucose levels may not instigate direct damage to proximal tubules. High glucose might reduce the NAD/NADH ratio, which causes a reduction in sirtuin activity. In that case, the sirtuin promoter activity might also be downregulated via an autofeedback mechanism [21]. Our novel findings indicate that GLUT2 functions as a signal sensor to induce importin-α1/HNF1α in proximal tubules, leading to the induction of SGLT2. Upregulated SGLT2 might in turn reduce SIRT1 levels. In conclusion, reduced SIRT1 in proximal tubules leading to a reduction in SIRT1 in podocytes might be a novel diagnostic marker and therapeutic target in DKD.
